# Outlines of Rural Hygiene

**Published:** 1898-04

**Authors:** 


OUTLINES OF RURAL HYGIENE. For Physicians, Students and Sanitarians. By HARVEY B. BASHORE, M. D., Inspector for the State Board of Health of Pennsylvania. With an appendix on The Normal Distribution of Chlorine, by Prof. Herbert E. Smith, of Yale University. Illustrated with twenty engravings, pp. vi.—84. Philadelphia, New York, Chicago: The F. A. Davis Co., Publishers.

This little book of seventy-odd pages is one whose value is as great as its pages are few. It deals in a simple and entirely practical manner with the problems which confront the sanitarian in country districts. The two most important items for the consideration of the country householder—the water supply and waste disposal—are handled in such manner that anyone availing himself of the help of this book may with moderate outlay make his dwelling a sanitary one in these respects. The soil, the relation of ground-air and ground-moisture to the healthfulness of localities and dwellings, house construction with reference to ground-air and ground moisture, the ventilation of dwellings, school hygiene, and the disposal of the dead are other topics considered in this work. The book should be in the possession of every suburban and country physician and in every village library. Indeed, teachers' classes and advanced physiology classes in village high schools might very properly include such a work on hygiene in their courses of study. M. J. F.

